# The impact of emphysema on dosimetric parameters for stereotactic body radiotherapy of the lung

**DOI:** 10.1093/jrr/rrw060

**Published:** 2016-09-30

**Authors:** Satoru Ochiai, Yoshihito Nomoto, Yasufumi Yamashita, Tomoki Inoue, Shuuichi Murashima, Daisuke Hasegawa, Yoshie Kurita, Yui Watanabe, Yutaka Toyomasu, Tomoko Kawamura, Akinori Takada, Noriko, Shigeki Kobayashi, Hajime Sakuma

**Affiliations:** 1 Department of Radiation Oncology, Matsusaka Central Hospital, 102 Kobou Kawai-machi, Matsusaka, Mie 515-8566, Japan; 2 Department of Radiology, Mie University School of Medicine, 2-174 Edobashi, Tsu, Mie 514-8507, Japan; 3 Department of Radiology, Matsusaka Central Hospital, 102 Kobou Kawai-machi, Matsusaka, Mie 515-8566, Japan

**Keywords:** lung cancer, stereotactic body radiation therapy, stereotactic ablative radiation therapy, emphysema, chronic obstructive pulmonary disease, homogeneity index, conformity index, dosimetric parameter

## Abstract

The purpose of this study was to evaluate the impact of emphysematous changes in lung on dosimetric parameters in stereotactic body radiation therapy (SBRT) for lung tumor. A total of 72 treatment plans were reviewed, and dosimetric factors [including homogeneity index (HI) and conformity index (CI)] were evaluated. Emphysematous changes in lung were observed in 43 patients (60%). Patients were divided into three groups according to the severity of emphysema: no emphysema (*n =* 29), mild emphysema (*n =* 22) and moderate to severe emphysema groups (*n =* 21). The HI (*P <* 0.001) and the CI (*P =* 0.029) were significantly different in accordance with the severity of emphysema in one-way analysis of variance (ANOVA). The HI value was significantly higher in the moderate to severe emphysema group compared with in the no emphysema (Tukey, *P <* 0.001) and mild emphysema groups (*P =* 0.002). The CI value was significantly higher in the moderate to severe emphysema group compared with in the no emphysema group (*P =* 0.044). In multiple linear regression analysis, the severity of emphysema (*P <* 0.001) and the mean material density of the lung within the PTV (*P <* 0.001) were significant factors for HI, and the mean density of the lung within the PTV (*P =* 0.005) was the only significant factor for CI. The mean density of the lung within the PTV was significantly different in accordance with the severity of emphysema (one-way ANOVA, *P =* 0.008) and the severity of emphysema (*P <* 0.001) was one of the significant factors for the density of the lung within the PTV in multiple linear regression analysis. Our results suggest that emphysematous changes in the lung significantly impact on several dosimetric parameters in SBRT, and they should be carefully evaluated before treatment planning.

## INTRODUCTION

Surgical resection is the gold standard therapy for Stage I non–small cell lung cancer (NSCLC). However, only 10–15% of patients benefit from surgical intervention because of comorbidity, such as poor lung function [1]. Stereotactic body radiation therapy (SBRT) provides promising local control and survival benefit for Stage I NSCLC patients, especially medically inoperable cases [2, 3]. Tobacco smoking is the most important cause of lung cancer, and it is also the main cause of chronic obstructive pulmonary disease (COPD) [4, 5]. According to a number of studies, about half of the patients who are treated with SBRT for lung tumor have COPD [6–8].

Emphysema is defined as a permanent enlargement of the airspace distal to the terminal bronchioles, with destruction of the alveolar wall and loss of alveolar attachments. Emphysematous changes were observed as low attenuation areas on computed tomography (CT) [9]. Although the low attenuation value could well affect the dose distribution for SBRT for lung tumor, it appears that this has not been well studied to date.

This retrospective analysis was conducted to evaluate the impact of emphysematous changes in the lung on dosimetric parameters for SBRT for lung tumor.

## MATERIALS AND METHODS

### Study design and patient selection

This is a single institutional retrospective study of dosimetric parameters in treatment planning for SBRT for lung tumor. The analysis was conducted after approval by our institutional review board. Informed consent had been obtained from all patients for use of their data in medical records and radiation treatment planning systems (RTPSs) for clinical research. Written informed consent to be included in this study was waived.

Between October 2009 and September 2015, a total of 120 patients who were treated with SBRT for lung tumor were reviewed. The eligibility criteria of this study were as follows: (i) histologically or cytologically confirmed NSCLC; (ii) a pulmonary nodule that had been clinically diagnosed as primary lung cancer by pulmonologists and radiologists based on clinical course and radiologic findings of computed tomography (CT) and 18F-fluorodexoxy glucose (FDG) positron emission tomography (PET): (iii) pretreatment imaging study of chest with high-resolution CT (HRCT) with 1.25 mm slice thickness. The exclusion criteria were as follows: (i) pulmonary nodule(s) diagnosed as metastatic cancer based on clinical course of patients (*n =* 10); (ii) multiple targets treated at the same time (*n =* 2); (iii) prior thoracic radiotherapy (including SBRT to other NSCLC or pulmonary nodule (*n =* 4); (iv) dose fractionation of SBRT other than 48 Gy in 4 fractions (*n =* 32). We essentially prescribed a total dose of 48 Gy in 4 fractions for a peripherally located tumor. Other dose fractionations, such as 60 Gy in 8–10 fractions, were chosen in patients who had targets near critical organs, such as a main bronchus or the esophagus. In such cases, shapes of beams were often modified to avoid the critical organ, which might affect the dosimetric parameters of targets. The evaluation of the effect according to the distance from target to critical organs was difficult. As a result, a total of 72 patients were eligible and included in this analysis.

### Assessment of emphysematous changes in lung

On HRCT, pulmonary emphysema lesions appear as low attenuation areas (LAAs). Each LAA can be distinguished from the normal lung, and it is characterized by not having a capsule. The severity of emphysema was evaluated by the Goddard classification [10]. The Goddard classification is a visual scale in which the area of vascular disruption and the low attenuation value are scored for each lung field. Zero represents no abnormality; 1 is given for up to 25%, 2 for up to 50%, 3 for up to 75%, and 4 for total involvement or almost total absence of normal lung tissue. There is a possible score of 24 as a maximum for each patient. Patients with 8 points or less were classified as mild, 9 to 16 points as moderate, and more than 16 points as severe. HRCT scans were assessed by three radiologists (Y.K., D.H. and S.M.) and scored (using the Goddard classification) after consultation. The patients were divided into three groups: the no emphysema, mild emphysema, and moderate to severe emphysema groups.

### SBRT

Our methods for treatment planning have previously been described in detail [11, 12]. SBRT plans were generated using the Pinnacle planning system (Phillips Medical System, Andover, MA). Monitor units were calculated using a collapsed cone convolution algorithm. The clinical target volume was defined as the visible gross tumor volume (GTV). The internal target volume (ITV) was chosen considering CT using a slow scan technique. The planning target volume (PTV) was defined as the ITV with a 5-mm margin to allow for set-up uncertainty. A respiration-monitoring apparatus was used for breath-holding conditions if the amplitude of the tumor motion was large. In the case where the amplitude of the tumor motion was within 15 mm in each direction, instead of using the breath-holding technique, the range of the tumor was considered in the delineation of the ITV. The PTV was defined as the ITV plus 5 mm of set-up margin. The radiation ports were set to the PTV with a 5-mm margin, except in one patient (3-mm margin). All patients were treated using 3D conformal radiation therapy with multiple static ports (7–13 ports). The dose was prescribed to the isocenter. The patients included in this study were irradiated with a total dose of 48 Gy in four fractions. These definitions of targets and techniques basically followed the protocol of the Japanese Clinical Oncology Group (JCOG) 0403 study [13, 14]. Irradiation was performed using 6-MV photons with a conventional flattening filter from an Elekta Synergy linear accelerator (Elekta, Stockholm, Sweden). Daily online cone-beam CT-based volumetric image-guided radiation therapy using soft-tissue target registration was applied immediately prior to any SBRT.

### Contouring of organs at risk and the definition of lung

Lung contours were created using Pinnacle software for automatic contouring and edited manually to follow the visible lung borders. Other organs at risk (OARs), including aorta, esophagus, trachea/bronchus, heart and spinal cord were contoured manually. The volume of total lung was defined as the sum of right and left lung minus the PTV. The volume of pulmonary tissue within the PTV was defined as the lung within the PTV, minus the GTV.

### Evaluation parameters

In dosimetric evaluation of the PTV, maximum (D-max), mean (D-mean) and minimum (D-min) dose to the PTV, D2, D5, D50, D95 and D98 were evaluated—where D2, D5, D50, D95 and D98 were the minimum dose received by 2%, 5%, 50%, 95% and 98% of the PTV, respectively. The homogeneity index (HI) was defined as the ratio of the maximum dose in the PTV to the minimum dose in the PTV [15]. The conformity index (CI) was defined as the ratio of the volume of the isodose shell that provided coverage of 95% of the PTV volume [16]. For OARs, the analysis included D1 cm^3^ and D10 cm^3^, where D*x* cm^3^ is the minimum dose received by *x* cm^3^ of the organ (aorta, esophagus, trachea/bronchus, heart). For the spinal cord, the maximum dose was analyzed according to the JCOG 0403 protocol [14]. The mean dose and set of appropriated V*x* values were used to assess the lung; V*x* was the volume of the organ that received a dose of *x* Gy or more. For example, V20 was the volume of organ receiving a dose of 20 Gy or more. For the PTV and the total lung, the mean dose–volume histograms (DVHs) were calculated from the tabular DVH data of all patients at 100 cGy resolution. The main objective parameters in this study were the HI and the CI of the PTV. The secondary objective parameter was the V20 of total lung.

### Estimation of the material density of the lung

The material densities of the total lung and pulmonary tissue within the PTV were estimated from the mean CT number, using the CT number to material density conversion table of Pinnacle.

### Comparison of virtual treatment plans

A virtual spherical GTV with a maximum diameter of 20 mm was created in the right lower lobe of lung on the CT of a healthy volunteer. A virtual PTV was created as the virtual GTV with a 15-mm margin in each direction. The isocenter was set to the center of the PTV. The regions of interest (ROIs) of the lung within the PTV and the total lung were created in the treatment plan. The material density of the GTV was overridden as 1.00 g/cm^3^ using Pinnacle software. The material densities of the total lung and the lung within the PTV were overridden with the mean value of the no emphysema (‘No emphysema model’), mild emphysema (‘Mild emphysema model’), and moderate to severe emphysema (‘Moderate to severe model’) groups in our cohort. Beams were shaped into the PTV plus a 5-mm margin, and 12 static ports were created. A total dose of 48 Gy was administered in four fractions, and the dose was prescribed to the isocenter. Dose distribution on the axial image and the DVH of the PTV and the total lung were compared. The DVHs were calculated from the tabular DVH data at 100 cGy resolution.

### Statistical analysis

One-way analysis of variance (ANOVA) was used in comparing the three groups, and Tukey's test was used as a *post-hoc* test. The Jonckheere–Terpstra test were conducted to assess the trends among groups. The mean values of each factor were compared for each two groups using Student's *t* test. For continuous variables, the threshold values for Student's *t* test were the medians. Severity of emphysema and variables with a *P* value of <0.20 in Student's *t* test were included in multiple linear regression analysis, using a step-wise method. Spearman's rank correlation coefficient was used to evaluate the relationship between variables. A *P* value of <0.05 was deemed to be statistically significant. All statistical analyses were performed using EZR (Saitama Medical Center, Jichi Medical University, The R Foundation for Statistical Computing) [17].

## RESULTS

### Patients

Between October 2009 and September 2015, 120 patients with 123 lung tumors were treated with SBRT at our institution. Among them, 72 patients (60.0%) with 72 tumors (56%) were included in this study. The patient characteristics are shown in Table 1.

**Table 1.** Patient characteristics

Total number of patients	72
Gender
Male (%)	44 (61)
Female (%)	28 (39)
Age (years)
Median (range)	81 (46–91)
Mean (SD)	78 (8.3)
Previous surgery for another NSCLC	
Yes (%)	16 (22)
No (%)	56 (78)
Emphysematous change	
Yes (%)	43 (60)
No (%)	29 (40)
Severity of emphysema (Total Goddard score)	
Mild (1–8)	22 (31)
Moderate (9–16)	11 (15)
Severe (17–24)	10 (14)
Mean CT number of total lung
Median (range)	292.2 (158.2–437.8)
Mean (SD)	298.5 (66.7)
Mean CT number of pulmonary tissue within PTV	
Median (range)	291.7 (73.6–512.8)
Mean (SD)	299.2 (101.1)
Pathologically confirmed	
Yes (%)	33 (46)
No (%)	39 (54)
Location of target	
Right	46 (64)
Left	26 (36)
Upper/middle	41 (57)
Lower	31 (43)
Maximum diameter of GTV (mm)	
Median (range)	18 (7–45)
Mean (SD)	20 (7.6)
Planning target volume (cm^3^)	
Median (range)	49.34 (22.77–188.00)
Mean (SD)	61.62 (34.91)

NSCLC = non–small cell lung cancer, CT = computed tomography, PTV = planning target volume, GTV = gross tumor volume, SD = standard deviation.

### Emphysematous changes in lung

Emphysematous changes in lung were observed in 43 patients (60%). Among patients with emphysema, 22 patients (31%) were classified as mild, 11 patients (15%) as moderate, and 10 patients (14%) as severe groups, respectively (Table 1). Patients were divided into three groups in accordance with the severity of emphysema: no emphysema (*n =* 29), mild emphysema (*n =* 22), and moderate to severe emphysema (*n =* 21) groups.

### Comparison of dosimetric parameters according to severity of emphysema

**Fig. 1.**
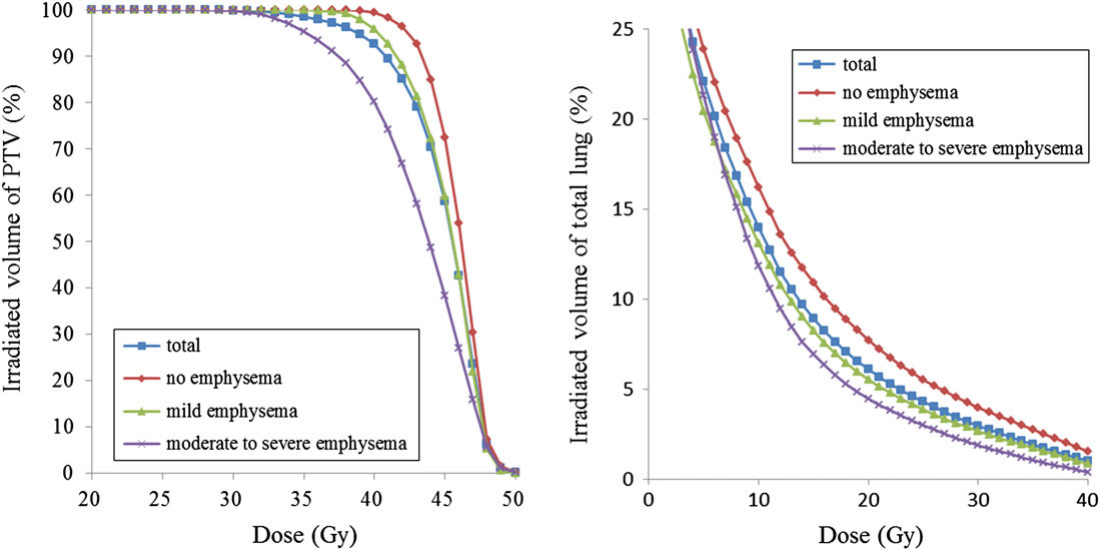
Mean DVH of PTV and total lung.

The results of the evaluated parameters are shown in Table 2, and the parameters were compared in accordance with severity of emphysema. The mean DVH of the PTV and of the total lung are shown in Fig. 1.

**Table 2.** Comparison of dosimetric parameters according to severity of emphysema

Metrics	Unit	Total (*n =* 72)	No emphysema (*n =* 29)	Mild (*n =* 22)	Moderate to severe (*n =* 21)	*P* value (O-A)
Mean PTV D-max (SD)	Gy	48.85 (0.88)	48.87 (0.92)	48.68 (0.62)	49.00 (1.06)	0.494
Mean PTV D2 (SD)	Gy	48.18 (0.97)	48.38 (0.75)	48.18 (0.69)	47.91 (1.37)	0.236
Mean PTV D5 (SD)	Gy	47.76 (1.23)	48.11 (0.75)	47.85 (0.82)	47.16 (1.81)	0.749
Mean PTV D50 (SD)	Gy	45.23 (2.30)	46.33 (0.84)	45.52 (1.53)	43.41 (3.17)	<0.001
Mean PTV D-mean (SD)	Gy	45.05 (2.21)	46.08 (0.90)	45.38 (1.53)	43.29 (2.98)	<0.001
Mean PTV D95 (SD)	Gy	41.40 (3.03)	43.04 (1.40)	41.69 (2.10)	38.85 (3.80)	<0.001
Mean PTV D98 (SD)	Gy	40.39 (3.13)	40.39 (3.13)	42.13 (1.58)	37.80 (3.85)	<0.001
Mean PTV D-min (SD)	Gy	37.44 (3.40)	39.54 (1.87)	37.37 (2.75)	34.61 (3.65)	<0.001
Conformity index (SD)		1.52 (0.20)	1.48 (0.17)	1.48 (0.15)	1.61 (0.25)	0.029
Homogeneity index (SD)		1.32 (0.13)	1.24 (0.07)	1.31 (0.11)	1.43 (0.15)	<0.001
Mean total lung V5 (SD)	%	22.05 (6.62)	23.87 (6.20)	20.42 (7.35)	21.26 (6.07)	0.149
Mean total lung V10 (SD)	%	13.95 (5.12)	16.19 (4.63)	13.09 (5.47)	11.77 (4.29)	0.005
Mean total lung V15 (SD)	%	8.93 (3.68)	10.89 (3.71)	8.26 (3.05)	6.93 (2.95)	<0.001
Mean total lung V20 (SD)	%	6.10 (2.76)	7.73 (2.86)	5.51 (1.92)	4.47 (2.16)	<0.001
Mean total lung V30 (SD)	%	2.97 (1.64)	3.98 (1.72)	2.67 (1.11)	1.89 (1.12)	<0.001
Mean total lung V40 (SD)	%	1.00 (0.88)	1.53 (0.97)	0.85 (0.65)	0.41 (0.43)	<0.001
Mean lung dose (SD)	Gy	4.18 (1.26)	4.79 (1.22)	3.87 (1.17)	3.68 (1.10)	0.002

*n* = number of patients, O-A = one-way analysis of variance, PTV = planning target volume, SD = standard deviation.

**Fig. 2.**
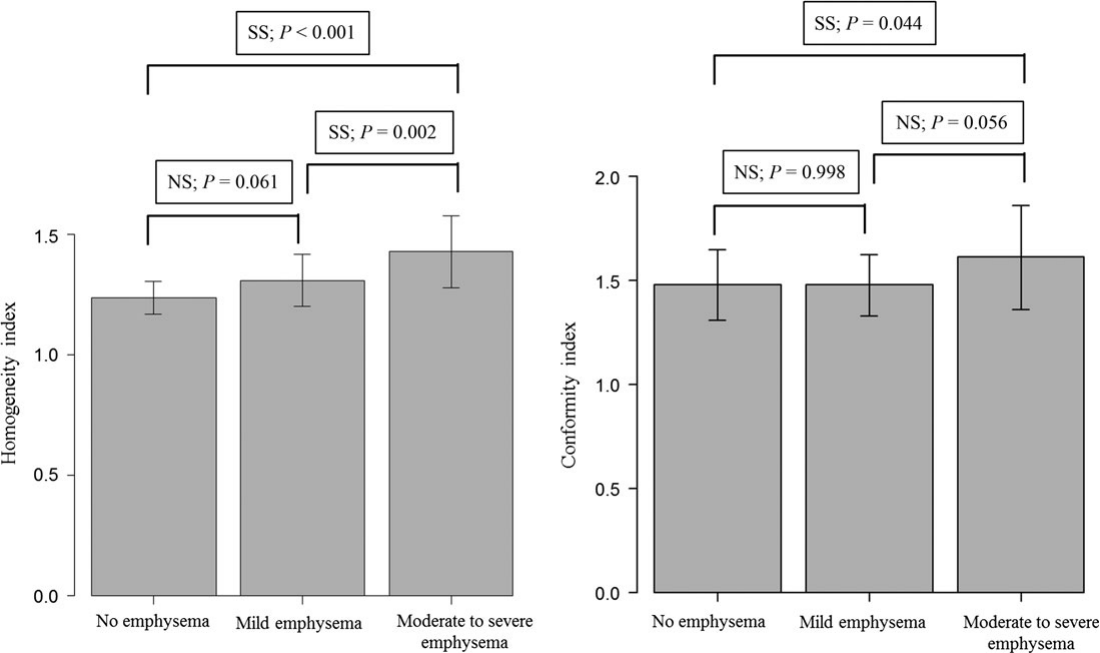
Comparison of homogeneity index and conformity index between no emphysema, mild emphysema, and moderate to severe emphysema groups. SS = statistically significant, NS = not significant.

For the parameters of the PTV, there were significant differences in accordance with severity of emphysema in D50, D-mean, D95, D98 and D-min (one-way ANOVA, *P <* 0.01). There were significant differences in HI and CI (one-way ANOVA, *P <* 0.05). HI value was significantly higher in the moderate to severe emphysema group compared with in the no emphysema group (Tukey, *P <* 0.001) and compared with the mild emphysema group (Tukey, *P =* 0.002), respectively (Fig. 2). There was an increasing trend in HI in accordance with the severity of emphysema (Jonckheere–Terpstra test, *P <* 0.001). The CI value was significantly higher in the moderate to severe group compared with that in no emphysematous change group (Turkey, *P =* 0.044) (Fig. 2). There was an increasing trend in CI (Jonckheere–Terpstra test, *P =* 0.021) in accordance with severity of emphysema.

**Fig. 3.**
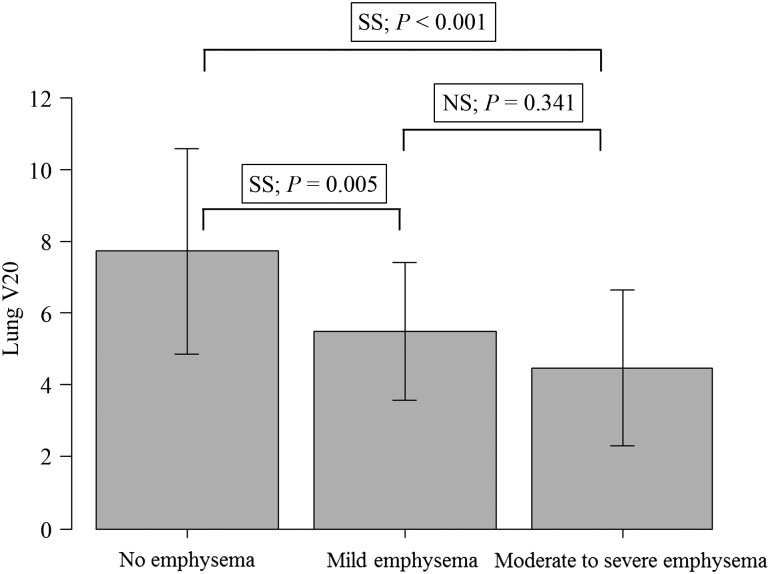
Comparison of lung V20 between no emphysema, mild emphysema, and moderate to severe emphysema groups. SS = statistically significant, NS = not significant.

For the parameters of the total lung, there were significant differences in accordance with severity of emphysema in V10, V15, V20, V30 and V40 (one-way ANOVA, *P <* 0.05) (Table 2). The lung V20 was significantly lower in the moderate to severe emphysema group compared with in the no emphysema group (Tukey, *P <* 0.001), and lower in the mild emphysema group compared with in the no emphysema group (Tukey, *P =* 0.005). There was a decreasing trend in lung V20 in accordance with severity of emphysema (Jonckheere–Terpstra test, *P <* 0.001) (Fig. 3).

For the irradiated dose to OARs other than the total lung, the results are summarized in Table 3. These parameters were not significantly different in accordance with severity of emphysema.

**Table 3.** Irradiated dose to organ at risk

Organ	Metrics	Unit		Total (*n =* 72)
Spinal cord				
	D-max	Gy	Median (range)	4.69 (0.43–16.70)
Trachea/bronchus				
	D10 cm^3^	Gy	Median (range)	1.94 (0.07–17.20)
Esophagus				
	D1 cm^3^	Gy	Median (range)	4.24 (0.18–21.88)
	D10 cm^3^	Gy	Median (range)	2.01 (0.15–15.20)
Pulmonary artery				
	D1 cm^3^	Gy	Median (range)	12.71 (0.45–47.20)
	D10 cm^3^	Gy	Median (range)	4.11 (0.18–44.50)
Aorta				
	D1 cm^3^	Gy	Median (range)	12.51 (0.48–44.51)
	D10 cm^3^	Gy	Median (range)	9.65 (0.25–27.74)
Heart				
	D1 cm^3^	Gy	Median (range)	10.55 (0.23–45.30)
	D10 cm^3^	Gy	Median (range)	6.63 (0.21–36.26)

*n* = number of patients, D-max = maximum dose, D*x* cm^3^ = minimum dose received by *x* cm^3^ of organ at risk.

### Comparison of other variables

The results of Student's *t* test for other variables are shown in Table 4. HI was significantly different in accordance with gender (*P =* 0.007), mean material density of total lung (*P <* 0.001), and mean material density of pulmonary tissue within the PTV (*P <* 0.001). CI was significantly different in accordance with the volume of the PTV (*P =* 0.005), and mean pulmonary tissue within the PTV (*P =* 0.011). Lung V20 was significantly different in accordance with gender (*P <* 0.001), age (*P =* 0.048), maximum tumor diameter (*P =* 0.011), volume of PTV (*P <* 0.001), mean material density of total lung (*P <* 0.001), and mean material density of pulmonary tissue within the PTV (*P =* 0.020).

**Table 4.** Results of Student's *t* test

Variable	Mean HI (SD)	*P*	Mean CI (SD)	*P*	Mean lung V20 (SD)	*P*
Gender		0.007		0.207		<0.001
Male	1.35 (0.14)		1.54 (0.21)		5.06 (2.09)	
Female	1.26 (0.11)		1.48 (0.17)		7.73 (2.91)	
Age		0.756		0.417		0.048
≤81	1.31 (0.14)		1.53 (0.21)		5.44 (2.57)	
>81	1.32 (0.13)		1.50 (0.19)		6.72 (2.82)	
Prior surgery		0.746		0.713		0.070
Yes	1.32 (0.13)		1.50 (0.14)		6.41 (2.95)	
No	1.31 (0.15)		1.52 (0.21)		5.00 (1.55)	
Tumor location		0.802		0.898		0.367
Upper/middle	1.32 (0.14)		1.51 (0.22)		6.36 (3.01)	
Lower	1.31 (0.13)		1.52 (0.17)		5.76 (2.39)	
		0.857		0.434		0.218
Right	1.32 (0.14)		1.50 (0.21)		6.40 (2.58)	
Left	1.31 (0.12)		1.54 (0.17)		5.56 (3.02)	
Maximum diameter of GTV (mm)		0.472		0.394		0.011
≤18	1.33 (0.16)		1.53 (0.19)		5.44 (2.17)	
>18	1.30 (0.09)		1.49 (0.21)		6.96 (3.10)	
Volume of PTV (cm^3^)		0.67		0.005		<0.001
≤49.3	1.31 (0.12)		1.58 (0.23)		4.76 (1.75)	
>49.3	1.32 (0.15)		1.45 (0.13)		7.44 (2.94)	
Density of total lung (g/cm^3^)		<0.001		0.117		<0.001
≤0.20	1.38 (0.14)		1.55 (0.24)		5.01 (2.17)	
>0.20	1.23 (0.07)		1.47 (0.12)		7.46 (2.83)	
Density of lung within PTV (g/ cm^3^)		<0.001		0.011		0.020
≤0.20	1.39 (0.14)		1.57 (0.24)		5.41 (2.36)	
>0.20	1.23 (0.07)		1.45 (0.11)		6.92 (2.99)	

HI = homogeneity index, CI = conformity index, lung V20 = volume of total lung receiving 20 Gy or more, Prior surgery = prior surgery for another non–small cell lung cancer, PTV = planning target volume, Density = estimated mean material density.

### Multiple linear regression analysis

In evaluation of HI, severity of emphysema, gender, mean material density of pulmonary tissue within the PTV, and mean material density of the total lung were included as variables for the multiple linear regression analysis. Of these, there was strong correlation between severity of emphysema and the mean material density of the total lung (Spearman's *rho =* 0.707, *P <* 0.001). Although there were significant correlations between severity of emphysema and mean material density of the total lung (*P <* 0.001) and gender (*P <* 0.001), these correlations were moderate (Spearman's *rho*: 0.415 and 0.556). We selected severity of emphysema as a variable for further evaluation and excluded mean material density of the total lung from the variables. In multiple linear regression analysis using a step-wise method, severity of emphysema (*P <* 0.001) and mean material density of pulmonary tissue within the PTV (*P <* 0.001) were selected as significant factors for HI (*F =* 40.94, *P <* 0.001, *R*^2^ *=* 0.529). In evaluation of CI, severity of emphysema, volume of the PTV, and mean material densities of the pulmonary tissue within the PTV and the total lung were included as variables for the analysis. We selected severity of emphysema as a variable for further evaluation and excluded mean material density of the total lung from the variables. In multiple linear regression analysis, mean material density of the total lung (*P <* 0.001) was selected as the only significant factor for CI (*F =* 8.39, *P =* 0.005, *R*^2^ *=* 0.094).

In evaluation of lung V20, severity of emphysema, gender, age, prior surgery for another NSCLC, maximum tumor diameter, volume of the PTV, mean material density of pulmonary tissue within the PTV and mean material density of the total lung were included as variables for the analysis. There was strong correlation between maximum tumor diameter and the volume of the PTV (Spearman's *rho =* 0.795, *P <* 0.001). We selected severity of emphysema and volume of the PTV for further evaluation and excluded mean material density of the total lung and maximum tumor diameter from the variables. In multiple linear regression analysis, severity of emphysema (*P =* 0.004), volume of the PTV (*P <* 0.001) and gender (*P <* 0.001) were significant factors for lung V20 (*F =* 38.79, *P <* 0.001, *R*^2^ *=* 0.592).

The results of the multiple linear regression analysis are shown in Table 5.

**Table 5.** Results of multiple linear regression analysis for homogeneity index, conformity index and lung V20

Variable		Coefficient (beta)	SE	*P* value	*F*	*R*^2^
Homogeneity index				<0.001	40.940	0.529
Intercept		1.400	0.034	<0.001		
Severity of emphysema	No/Mild/Moderate to severe	0.066	0.014	<0.001		
Density of lung within PTV	per g/cm^3^	–0.592	0.108	<0.001		
Conformity index				0.005	8.39	0.094
Intercept		1.656	0.053	<0.001		
Density of lung within PTV	per g/cm^3^	–0.596	–0.596	0.005		
Lung V20 (%)				<0.001	38.79	0.592
Intercept		5.376	0.511	<0.001		
Severity of emphysema	No/Mild/Moderate to severe	–0.899	0.302	0.004		
Volume of PTV	per cm^3^	0.044	0.006	<0.001		
Gender	Male/Female	1.903	0.51	<0.001		

SE = standard error, No = no emphysema, Mild = mild emphysema, Moderate to severe = moderate to severe emphysema, Density = estimated mean material density, PTV = planning target volume, Lung V20 = volume of total lung receiving 20 Gy or more.

### Correlation factors with material densities of the pulmonary tissue within the PTV and the total lung

There were significant differences in accordance with severity of emphysema in mean material densities of the total lung (one-way ANOVA, *P <* 0.001) and of the pulmonary tissue within the PTV (one-way ANOVA, *P =* 0.008). The results of the one-way ANOVA and Student's *t* test for the mean material density of the total lung and of the pulmonary tissue within the PTV are summarized in Table 6. The mean material density of the total lung was significantly higher in females compared with in males (*P <* 0.001). The mean material density of pulmonary tissue within the PTV was significantly higher in patients with a tumor in the lower lobe compared with in those with a tumor in upper or middle lobe (*P =* 0.016). For mean material density of total lung, severity of emphysema and gender were included as variables for multiple linear regression analysis using a step-wise method. Severity of emphysema (*P <* 0.001) and gender (*P =* 0.029) were selected as significant factors for mean material density of the total lung (*F =* 31.53, *P <* 0.001, *R*^2^ *=* 0.462) (Table 7). For mean material density of pulmonary tissue within the PTV, severity of emphysema and lobe of lung were included as variables for multiple linear regression analysis using a step-wise method. Severity of emphysema (*P <* 0.001) and lung lobe (*P <* 0.001) were selected as significant factors for mean material density of pulmonary tissue within the PTV (*F =* 12.60, *P <* 0.001, *R*^2^ *=* 0.246).

**Table 6.** Results of one-way ANOVA and Student's *t* test for densities of total lung and pulmonary tissue within PTV

Variable	Density of total lung (g/cm^3^) mean (SD)	*P* value	Density of pulmonary tissue within PTV (g/cm^3^) mean (SD)	*P* value
One-way ANOVA				
Severity of emphysema		<0.001		0.008
No emphysema	0.28 (0.11)		0.28 (0.11)	
Mild emphysema	0.21 (0.05)		0.23 (0.09)	
Moderate to severe emphysema	0.16 (0.04)		0.18 (0.10)	
Student's *t* test				
Gender		<0.001		0.596
Male	0.19 (0.05)		0.23 (0.11)	
Female	0.27 (0.07)		0.24 (0.10)	
Age (years)		0.231		0.326
≤81	0.23 (0.08)		0.25 (0.12)	
>81	0.21 (0.069)		0.22 (0.10)	
Prior surgery		0.786		0.422
Yes	0.22 (0.07)		0.25 (0.13)	
No	0.22 (0.07)		0.23 (0.10)	
Tumor location (lobe)		0.200		0.016
Upper	0.23 (0.08)		0.21 (0.09)	
Lower	0.21 (0.06)		0.27 (0.13)	
Tumor location (side)		0.839		0.737
Right			0.23 (0.10)	
Left			0.24 (0.12)	
Maximum tumor diameter (mm)		0.906		0.635
≤18	0.22 (0.08)		0.23 (0.12)	
>18	0.22 (0.07)		0.24 (0.09)	
Volume of PTV (cm^3^)		0.718		0.413
≤49.3	0.22 (0.07)		0.22 (0.10)	
>49.3	0.23 (0.07)		0.25 (0.11)	

Density = estimated mean material density, ANOVA = analysis of variance, Prior surgery = prior surgery for another non–small cell lung cancer, PTV = planning target volume.

**Table 7.** Results of multiple linear regression analysis for densities of total lung and pulmonary tissue within PTV

Variable		Coefficient (beta)	SE	*P* value	*F*	*R*^2^
Estimated mean material density of total lung (g/cm^3^)				<0.001	31.53	0.462
Intercept		0.284	0.010	<0.001		
Severity of emphysema	No/Mild/Moderate to severe	–0.046	0.009	<0.001		
Gender	Male/Female	0.034	0.015	0.029		
Estimated mean material density of lung within PTV (g/cm^3^)				<0.001	12.60	0.246
Intercept		0.251	0.018	<0.001		
Severity of emphysema	No/Mild/Moderate to severe	–0.058	0.014	<0.001		
Lobe of lung	Upper or middle/lower	0.083	0.234	<0.001		

SE = standard error, No = no emphysema, Mild = mild emphysema, Moderate to severe = moderate to severe emphysema.

### Comparison of virtual treatment plans

**Fig. 4.**
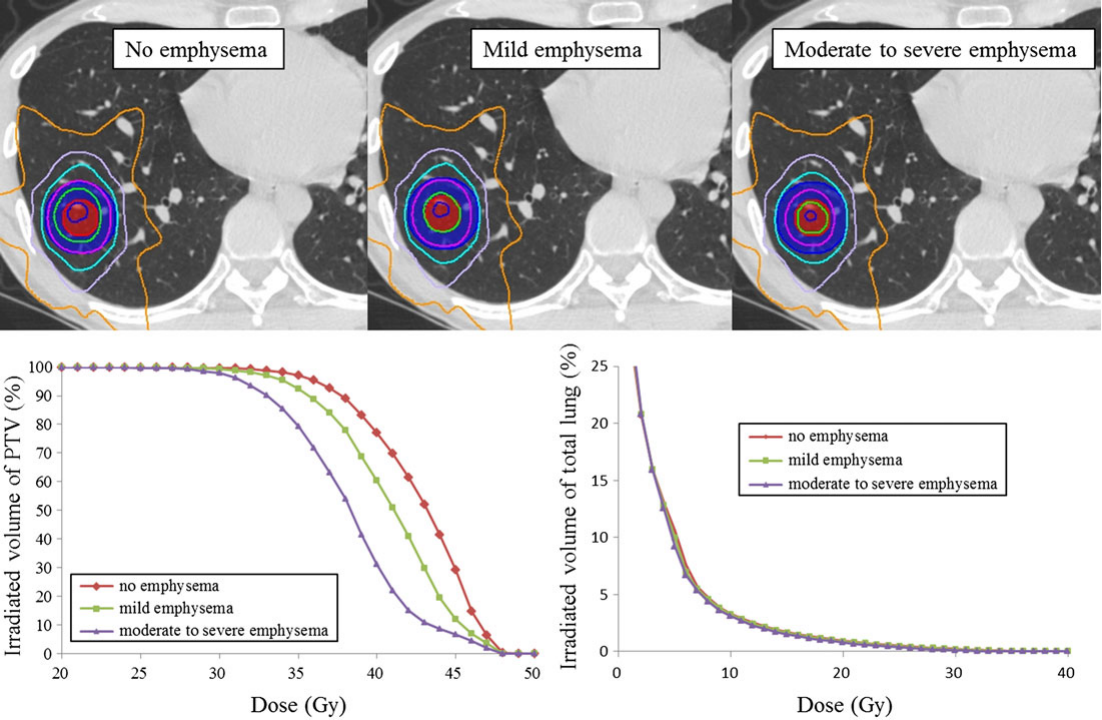
Comparison of virtual treatment plans. The material density of total lung was overridden as 0.28 g/cm^3^, 0.21 g/cm^3^ and 0.16 g/cm^3^ in no emphysema, mild emphysema, and moderate to severe emphysema models, respectively. The material density of pulmonary tissue within the PTV was overridden as 0.28 g/cm^3^, 0.23 g/cm^3^ and 0.18 g/cm^3^ in no emphysema, mild emphysema, and moderate to severe emphysema models, respectively. The material density of the GTV was overridden as 1.00 g/cm^3^ in every model.

Figure 4 shows a comparison of treatment plans between no emphysema, mild emphysema, and moderate to severe emphysema models. The material density of the total lung was overridden as 0.28 g/cm^3^, 0.21 g/cm^3^ and 0.16 g/cm^3^ in the no emphysema, mild emphysema, and moderate to severe emphysema models, respectively. The material density of pulmonary tissue within the PTV was overridden as 0.28 g/cm^3^, 0.23 g/cm^3^ and 0.18 g/cm^3^ in the no emphysema, mild emphysema, and moderate to severe emphysema models, respectively. Isodose lines of 48 Gy (blue), 45 Gy (green), 40 Gy (purple), 30 Gy (light blue), 20 Gy (lavender) and 10 Gy (orange) were represented on axial CT image. In the DVH of the PTV, the absorbed dose of PTV tended to decrease in accordance with increase in severity of emphysema. In the DVH of the total lung, although it was not obvious from the histogram, the absorbed dose tended to decrease in accordance with severity of emphysema in the data in the tabular DVH.

## DISCUSSION

In this study, we analyzed the effect of emphysematous changes in the lung on several dosimetric parameters of SBRT for lung cancer. Lung cancer and COPD are frequently caused by tobacco smoking [4, 5]. Thus, emphysematous changes in the lung can be often observed in patients with lung cancer. In our analysis, emphysematous changes in the lung were observed in 43 patients (60%), and the emphysematous changes in the lung were shown to affect several parameters significantly in the treatment planning for SBRT for lung tumor.

In this analysis, we estimated the material densities of the total lung and of the pulmonary tissue within the PTV from the mean CT number using the CT number to material density conversion table of RTPS. Severity of emphysema was significantly correlated with density of both the total lung and the pulmonary tissue within the PTV. Another significantly affecting factor for density of the total lung, and for pulmonary tissue within the PTV was lobe of lung. For the density of the total lung, the density decreased in accordance with increase in severity of emphysema and was higher in females compared with in males in our analysis. Gender has been reported as one of the factors influencing the lung density [18, 19]. Our results were compatible with these findings. The density of pulmonary tissue within the PTV decreased in accordance with increase in severity of emphysema and was higher in the lower lobe compared with in the upper or middle lobe in our analysis. It has been reported that lung density is higher in the posterior region compared with in the anterior region [20]. This difference could be explained by the effect of gravity. The lung volume of the posterior region is larger in the lower lobe compared with that of the upper or middle lobe, and the result of our analysis may have been affected by this difference.

Lung V20 is one of the dose constraint parameters used in treatment planning for lung cancer [21]. In our multivariate linear regression analysis, severity of emphysema was shown to be one of the significant factors for lung V20, and lung V20 decreased in accordance with increase in severity of emphysema (Table 2, Fig. 3). In several reports, patients with severe emphysema or COPD have been reported to have a low risk of severe radiation pneumonitis [6, 7]. Relatively lower absorption of dose in the lung region in patients with severe emphysema, due to lower density of the lung, might be one reason for relatively mild radiation pneumonitis in those patients.

There is no doubt that it is important to concentrate high-dose radiation on the target volume, while sparing normal adjacent tissue. Although there are a number of differences in the recommended definitions of HI and CI, several reports on SBRT for lung cancer recommend that dosimetric parameters including HI and CI be evaluated [16, 22, 23]. The HI tended to increase in accordance with increase in severity of emphysema, and the differences between the no emphysema and the moderate to severe groups, and between the mild emphysema and the moderate to severe groups were significant. In our multivariate linear regression analysis, severity of emphysema and mean material density of the pulmonary tissue within the PTV were shown to be significant factors. The CI also tended to increase in accordance with increase in severity of emphysema and the difference between the no emphysema and the moderate to severe emphysema groups was significant. In our multivariate linear regression analysis, the mean density of the pulmonary tissue within the PTV was the only significant factor for CI.

**Fig. 5.**
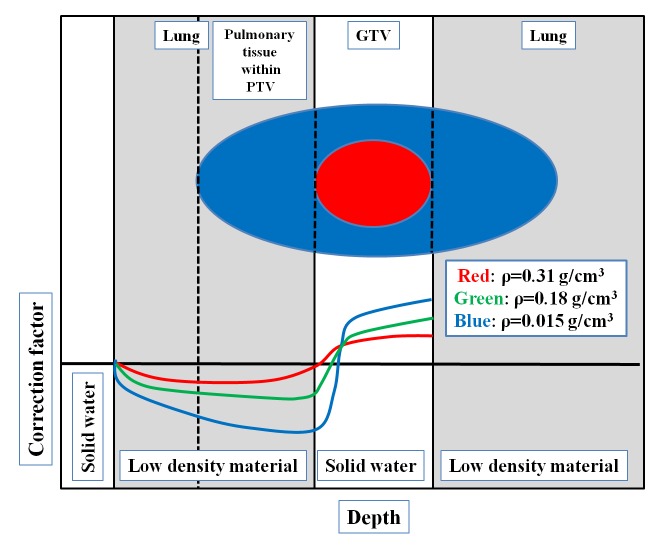
Schematic diagram of target in SBRT for lung cancer and correction factor in low-density material. The red oval represents the GTV and the blue oval represents the PTV in SBRT for lung cancer. The GTV is surrounded by a low-density-material region (pulmonary tissue within the PTV). The correction factor is decreasing within low-density material (gray area).

Rice *et al*. reported benchmark measurements for lung dose corrections for X-ray beams [24]. The correction factor, which was defined as the ratio of the dose in the heterogeneous phantom to the dose at the same point in the water phantom, decreased with decreasing density within the low-density material. In other words, the absorbed dose in the low-material-density area decreased with decreasing density within the low-density material. Figure 5 is a schematic diagram in which the correcting factors for various low-density materials are compared [24]. In the figure, the GTV, the PTV and the pulmonary tissue within the PTV in the setting of SBRT for lung tumor are also represented. The density of the pulmonary tissue within the PTV was decreasing in accordance with increase in severity of emphysema in our analysis, and this could result in decreasing D-min and D-95 of the PTV. The decrease of the absorbed dose in emphysematous region, where the material density is low, was considered to be the main reason for worsening of homogeneity and conformity in patients with emphysema.

Adding a wider margin in shaping the beams is one way to improve homogeneity for the target in patients with emphysema [25, 26]. However, that could result in poor conformity and increasing dose to the normal lung tissue. Although further investigation in the context of SBRT is needed, the use of relatively lower-energy photons, such as 4-MX, could improve the homogeneity and conformity at the target [22, 27, 28]. Another promising method for improving homogeneity and conformity is the use of modern radiation therapy techniques, such as volumetric-modulated arc radiotherapy [29]. In such cases, dose-prescription to a point seems to be unreliable [30], and dose–volume prescription, such as prescription to D50, D-mean or D95, would be favorable. However, our results suggest that the change from dose-prescription to isocenter to a dose–volume prescription would result in substantial dose escalation. The increased rate of absorbed dose by prescription to D50, D-mean and D95 was estimated to be approximately 6.1%, 6.5% and 15.9%, respectively. Moreover, in patients with emphysema, it was estimated to be approximately 7.9%, 8.2% and 1.1%, respectively. Thus, careful attention should be paid in case of dose escalation of SBRT with advanced technology, especially in patients with emphysematous change in the lung.

The retrospective nature of this study was an important limitation. In most of the patients, 5-mm margins were added to the PTV, and consideration about optimal margins in the shaping of beams was insufficient. Excluding patients with a so-called ‘centrally located tumor’ or a tumor located near a critical organ, such as the brachial plexus, was another limitation. The effect of the distance between a critical organ and the target was not considered. The relatively small cohort size was another limitation. Despite these study limitations, the results of this study will contribute to improving the quality of treatment planning for SBRT for lung tumor.

In conclusion, several parameters in treatment planning for SBRT for lung tumor were significantly affected by emphysematous changes in lung. Severity of emphysema was found to be the significant factor for HI and lung V20 in multiple linear regression analysis, and the mean material density of pulmonary tissue within the PTV, which is significantly correlated with severity of emphysema, was found to be the significant factor for CI. Thus, emphysematous changes in lung should be carefully evaluated before treatment planning.
